# Human electrophysiologic mapping of transaortic pacing targets for valve-integrated pacemakers

**DOI:** 10.1093/europace/euag266

**Published:** 2026-09-16

**Authors:** Iurii Karpenko, Andrei Ardashev, Jeremiah Wasserlauf, Evgeny Zhelyakov, Anna Lytchakov, Eric Rytkin, Igor R Efimov, Daniil P Aksenov

**Affiliations:** Odesa National Medical University, Department of Internal Medicine and Cardiovascular Pathology, Odesa, Ukraine; Endeavor Health Research Institute, Department of Radiology, 1033 University Pl, Suite 100, Evanston, IL 60201, USA; Endeavor Health Cardiovascular Institute, Department of Cardiology, Glenview, IL, USA; JSC ‘Medicina’, Department of Cardiology, Moscow, Russia; Feinberg School of Medicine, Northwestern University, Department of Radiology, Chicago, IL, USA; Northwestern University, 303 E Superior Str, SQ-11-529, Chicago, IL 60611, USA; Northwestern University, 303 E Superior Str, SQ-11-529, Chicago, IL 60611, USA; Endeavor Health Research Institute, Department of Radiology, 1033 University Pl, Suite 100, Evanston, IL 60201, USA; Northwestern University, 303 E Superior Str, SQ-11-529, Chicago, IL 60611, USA

**Keywords:** Transaortic pacing, Aortic cusps, His bundle, TAVR, Valve-integrated pacemaker

## Introduction

Conduction disturbances remain common after transcatheter aortic valve replacement (TAVR) and may require permanent pacing.^1,2^ Valve-integrated, millimetre-scale pacing systems could provide therapy at the site of valve implantation without a separate transvenous lead or intracardiac device.^3–5^ Their design depends on where a valve-adjacent electrode can achieve myocardial capture, the required output, and the resulting ventricular activation pattern. Pacing from the aortic cusps has been described during ventricular arrhythmia mapping,^6–10^ but systematic within-patient comparisons of potential valve-mounted pacing targets are limited. We therefore compared acute pacing characteristics at the left coronary cusp (LCC), right coronary cusp (RCC), non-coronary cusp (NCC), and LV His-adjacent region. Capture feasibility, current threshold, local electrogram amplitude, and paced QRS duration and morphology were assessed at each site.

## Methods

This prospective, single-centre feasibility study enrolled 25 consecutive patients undergoing clinically indicated electrophysiology study and radiofrequency ablation between March 2024 and July 2025. The protocol was approved by the local ethics committee, and all participants provided written informed consent. The transaortic pacing protocol was performed after completion of the clinical procedure. The four study sites were prespecified and examined systematically, independent of the preceding ablation target.

During sinus rhythm, a 4-mm non-irrigated catheter was advanced retrogradely across the aortic valve and positioned sequentially at the LCC, RCC, NCC, and LV His-adjacent region. Systemic anticoagulation with unfractionated heparin was maintained during left-sided instrumentation. Sites were localized using three-dimensional electroanatomic mapping, biplane fluoroscopy, and intracardiac echocardiography (*Figure 1*). The LV His-adjacent region was defined by projecting a right-sided His catheter to the left side of the septum on electroanatomic mapping and fluoroscopy. A local His potential was not required, and selective His-bundle capture was not adjudicated. Catheter contact was assessed from fluoroscopic stability, proximity to the mapped surface, and reproducibility of local electrograms.

**Figure 1:**
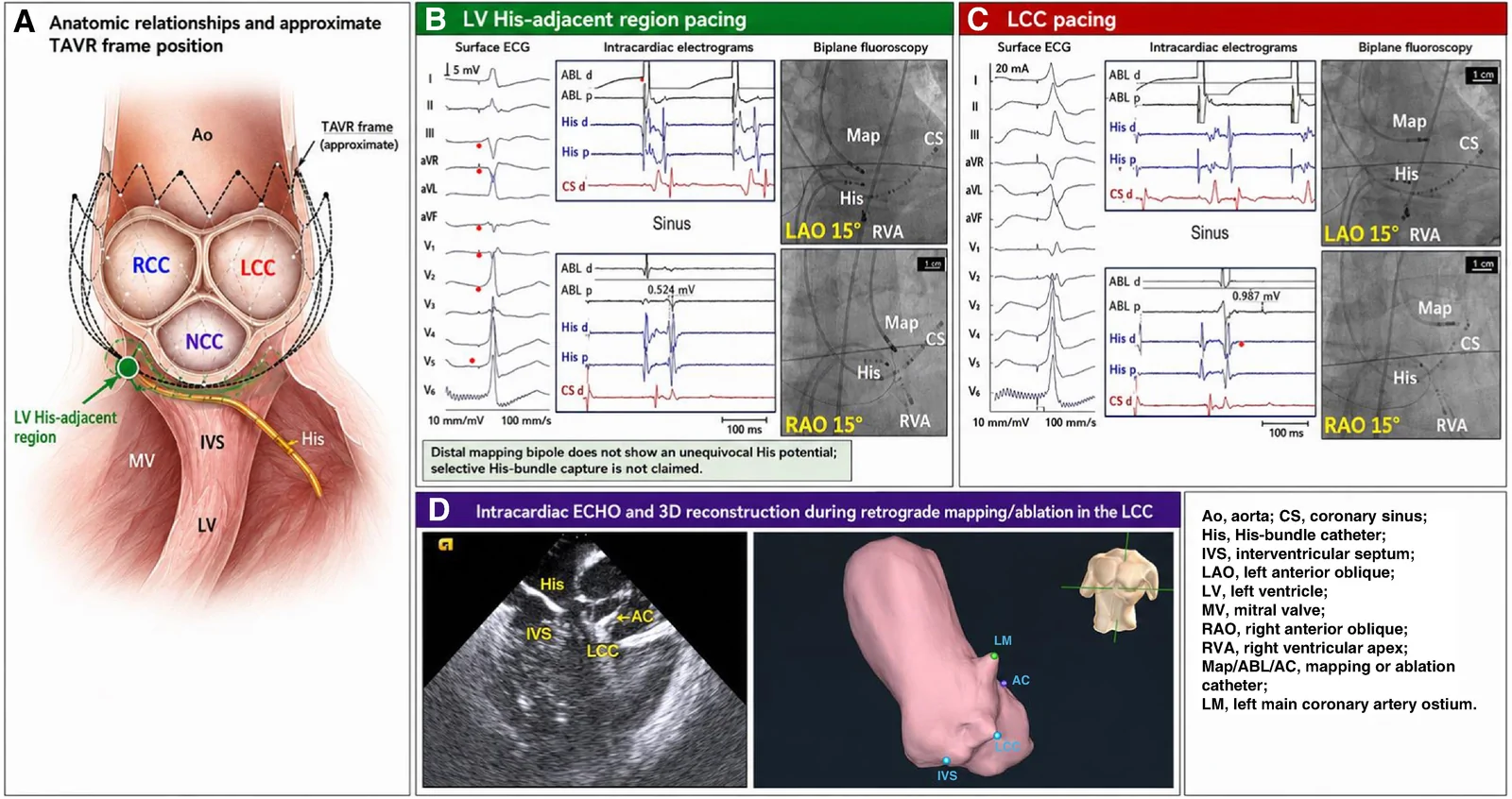
Anatomic localization and representative recordings. (*A*) Schematic relationships of the RCC, LCC, NCC, and LV His-adjacent region, with the approximate position of a TAVR frame. (*B*) Surface ECG, intracardiac electrograms, and biplane fluoroscopy during pacing from the anatomically defined LV His-adjacent region. The distal mapping bipole does not show an unequivocal His potential; selective His-bundle capture is not claimed. (*C*) Surface ECG, intracardiac electrograms, and biplane fluoroscopy during LCC pacing. (*D*) Intracardiac echocardiography showing the mapping/ablation catheter (AC) within the LCC. Ao, aorta; CS, coronary sinus; His, His-bundle catheter; IVS, interventricular septum; LAO, left anterior oblique projection; LV, left ventricle; Map/ABL/AC, mapping catheter; MV, mitral valve; RAO, right anterior oblique projection; RVA, right ventricular apex; TV, tricuspid valve.

Bipolar pacing was delivered from the distal pair at a 1-ms pulse width and a cycle length of 500–600 ms using constant-current stimulation. Output was reduced from 20 mA in 1-mA steps until loss of capture; the threshold was the lowest output producing eight consecutive captured beats with identical morphology. No capture was defined as failure at 20 mA and recorded as >20 mA without numerical imputation. Thresholds were summarized descriptively only among successful captures and were not included in the repeated-measures ANOVA. Two electrophysiologists independently assessed local electrogram amplitude, capture type, paced QRS duration, and lead V1 morphology; they resolved disagreements by consensus. Other continuous variables were compared by repeated-measures analysis of variance and categorical variables by Cochran's Q test, with Holm correction for multiple comparisons.

## Results

The cohort included 20 men and 5 women (mean age 52.1 ± 15.4 years); none had clinically significant aortic stenosis, and no substantial aortic valve calcification was documented. Clinical procedures addressed left-sided accessory pathways (*n* = 11), ventricular ectopy/tachycardia (*n* = 11), atrial tachycardia (*n* = 1), and AVNRT (*n* = 2). No RF energy was delivered at the assessed transaortic pacing sites; AVNRT ablations were performed from the right atrium within the triangle of Koch.

Ventricular capture was achieved in 25/25 patients at the LV His-adjacent region, 23/25 at the RCC, and 19/25 at the LCC, but in none at the NCC (overall *P* < 0.00001). NCC pacing produced atrial capture in 8/25 patients. Among successful ventricular captures, observed mean thresholds were 4.6 ± 2.1 mA at the LV His-adjacent region, 13.3 ± 5.1 mA at the RCC, and 14.2 ± 4.7 mA at the LCC. Paced QRS duration was also shortest at the LV His-adjacent region (137.5 ± 15.6 ms), vs. 165.5 ± 23.0 ms at the RCC and 164.1 ± 11.5 ms at the LCC (*P* < 0.00001). LV His-adjacent pacing most often produced an RBBB-like pattern in V1 (23/25), whereas RCC pacing was predominantly LBBB-like (22/23); LCC morphology was mixed. Mean ventricular electrogram amplitude was 1.8 ± 0.4 mV at the LV His-adjacent region, 1.4 ± 0.5 mV at the LCC, 1.3 ± 0.6 mV at the RCC, and 0.5 ± 0.3 mV at the NCC (*P* = 0.0011). No coronary injury, stroke, aortic perforation, worsening aortic regurgitation, or death occurred through 30 days. One groin haematoma resolved without intervention.

## Discussion

The LV His-adjacent region provided universal capture and, among successful captures, the lowest observed current threshold and shortest paced QRS. Coronary-cusp pacing was less reliable and required higher output when capture occurred. The lower LV His-adjacent threshold may reflect closer coupling to excitable septal myocardium and adjacent conduction tissue, consistent with its larger local ventricular electrogram, although selective His capture was not demonstrated.

The representative LV His-adjacent tracing contains a prominent atrial component on the proximal mapping bipole and no unequivocal distal His potential. We therefore interpret it as pacing from an anatomically defined His-adjacent region rather than proof of selective His-bundle capture. The inferior-lead polarity in this tracing and predominantly positive lead I during representative LCC pacing may reflect catheter orientation and the local myocardial exit site. Biplane fluoroscopy and intracardiac echocardiography support the assigned locations.

These findings should not be directly extrapolated to severe calcific aortic stenosis or TAVR, where calcification, frame/skirt geometry, implantation depth, orientation, chronic contact, and remodelling may alter sensing and capture. A valve-integrated LV His-adjacent electrode would require appropriate frame orientation and membranous-septal contact while avoiding skirt or calcific interposition. Objective contact force was not measured, and site-specific contact differences may have influenced thresholds. The mapping catheter does not reproduce implanted electrode geometry, fixation, or chronic tissue interface. Impedance was not systematically recorded; therefore, voltage and energy requirements could not be derived from current thresholds.

NCC pacing produced atrial capture in only 32%. A previous aortic cusp pacing series reported more frequent atrial capture from the NCC using a longer pulse width.^9^ Differences in catheter orientation, tissue contact, patient anatomy, and stimulation protocol may explain the lower rate. The present finding supports further study of the NCC as a possible atrial interface but does not establish a reliable dual chamber pacing strategy.

## Conclusion

Among four transaortic targets, the LV His-adjacent region showed the most favourable acute ventricular pacing profile. Validation in calcified and post-TAVR anatomy is required before these findings can guide valve-integrated pacing.

## Data Availability

The data underlying this article will be shared on reasonable request to the corresponding author.
